# Using genome editing to engineer universal platelets

**DOI:** 10.1042/ETLS20180153

**Published:** 2019-04-17

**Authors:** Moyra Lawrence, Annett Mueller, Cedric Ghevaert

**Affiliations:** 1 Department of Haematology, University of Cambridge and NHS Blood and Transplant, Long Road, Cambridge CB2 0PT, U.K.; 2 Wellcome Trust-Medical Research Council Cambridge Stem Cell Institute, Tennis Court Road, Cambridge CB2 1QR, U.K.

**Keywords:** genome editing, immune response, induced pluripotent stem cells, megakaryocytes, platelet adhesion and activation

## Abstract

Genome editing technologies such as zinc finger nucleases, TALENs and CRISPR/Cas9 have recently emerged as tools with the potential to revolutionise cellular therapy. This is particularly exciting for the field of regenerative medicine, where the large-scale, quality-controlled editing of large numbers of cells could generate essential cellular products ready to move towards the clinic. This review details recent progress towards generating HLA Class I null platelets using genome editing technologies for β2-microglobulin deletion, generating a universally transfusable cellular product. In addition, we discuss various methods for megakaryocyte (MK) production from human pluripotent stem cells and subsequent platelet production from the MKs. As well as simply producing platelets, differentiating MK cultures can enable us to understand megakaryopoiesis *in vivo* and take steps towards ameliorating bleeding disorders or deficiencies in MK maturation in patients. Thus by intersecting both these areas of research, we can produce optimised differentiation systems for the production of universal platelets, thus offering a stable supply of platelets for difficult-to-match patients and providing areas with transmissible disease concerns or an unpredictable supply of platelets with a steady supply of quality-controlled platelet units.

## Introduction

Although genome editing technologies such as zinc finger nucleases and TALENs (transcription activator-like effector nucleases) have been around for some time, it is the repurposing of the bacterial CRISPR/Cas9 defence system that has recently provided an efficient, affordable and adaptable method for rapid and reproducible gene editing [1,2]. This is particularly exciting for the field of regenerative medicine, where large-scale, quality-controlled editing of large numbers of cells could generate essential cellular products ready to move towards the clinic. In addition to being able to knock-specific genes out, the CRISPR/Cas9 system enables the insertion of relatively large constructs into cells [1,3], enabling the production of recombinant proteins from edited cells, or even more ambitiously, the production of cells for the targeted release of therapeutic proteins at specific sites in the body.

## Platelets and transfusions

Platelets are one of the few cell types which have no nucleus, which makes them one of the most ideal cell types for transfusion after gene editing. In addition, platelets retain their functionality after irradiation, reducing the likelihood of nucleated stem or progenitor cells surviving in the platelet unit. There is also evidence to suggest that mature megakaryocytes (MKs) can produce platelets effectively, even after irradiation [4]. Platelets could be one of the safest first-in-human stem cell-derived products for transfusion and provide us with an avenue to safely use gene editing methodology for the advent of new cellular products and medicines.

**Figure 1.**
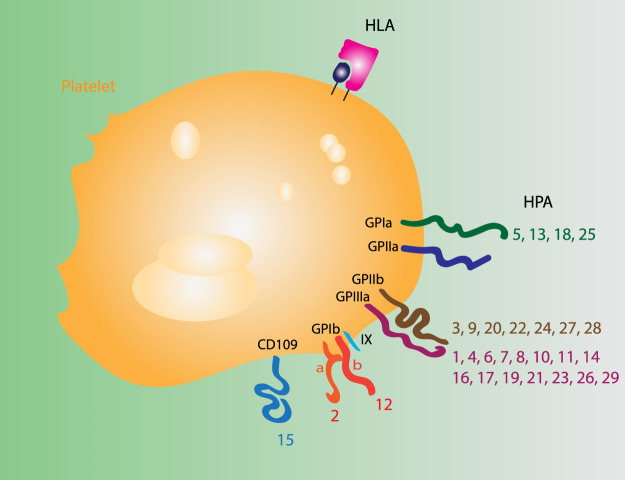
Immune-recognised platelet antigens. Antigens posing a risk of alloimmunisation after platelet transfusions.

Platelets are 2–4 µm anucleate discoid cells in the circulation [5,6] and are responsible for clotting. The deficiency of platelets is known as thrombocytopaenia and it can have diverse causes, including inherited and acquired bone marrow deficiency, chemotherapy, radiotherapy, trauma, major surgery, severe infection or hypercoagulation [7]. Patients with thrombocytopaenia are susceptible to severe bleeding events, and to prevent this they are transfused with donor platelets. Platelet units are generally ABO and Rhesus D matched [7,8] to avoid alloimmunisation against RhD from the few red cells present in the platelet unit or lysis from anti-A/anti-B present in the plasma into which platelets are suspended. However, some patients develop antibodies against antigens present on the surface of platelet themselves, namely HLA (histocompatibility leukocyte antigen) Class I and human platelet antigens (or in rare cases against auto- or drug-induced antigens) [9] (Figure 1). Among these, antibodies against HLA Class I are the most common [9–11]. Patients generally produce these antibodies as a response to previous transfusions or pregnancies. However, a certain proportion of individuals in the general population also have antibodies against other HLA Class I types. In one study, 17% of donors who had not been either pregnant or previously transfused had antibodies against HLA Class I [12], potentially posing a risk of rejection during standard platelet transfusion.

Providing matched platelet units for all thrombocytopoenic patients is difficult because platelet units have a very short shelf life of only 5–7 days at room temperature. There are times of year where matched units are difficult to find, and other times when units accumulate, wasting the resources used to collect and store them. The logistical challenge of providing HLA Class I-matched units for alloimmunised patients leads to significant costs, estimated to be in the region of five times the usual cost of platelet provision [9,13]. If platelets could be produced *in vitro* from ‘HLA Class I null’ seed cells, this would alleviate the need to match platelet units and provide a supply of universal platelets for transfusion.

## Megakaryocytes

**Figure 2.**
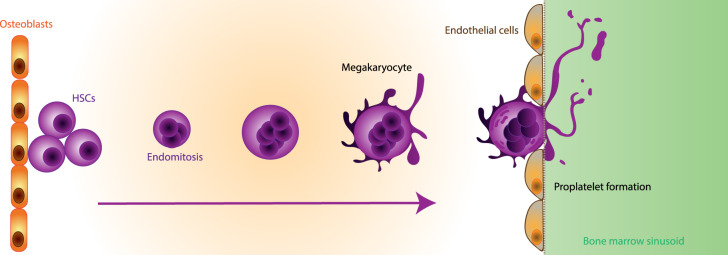
MK differentiation *in vivo*. Schematic of MK differentiation from HSCs in the bone marrow. As they differentiate, MKs move towards the bone marrow sinusoids where they adhere to endothelial cells and extend proplatelet extensions or entire cell fragments, budding platelets off into the circulation.

*In vivo* platelets are produced by multinucleated cells which are known as MKs [14]. These cells make up 0.01–0.03% of nucleated bone marrow cells [15] and are large cells of 5–50 µm in diameter with a mean diameter of 19.4 ± 3 µm [5,16]. MKs differentiate from haematopoietic stem cells (HSCs) in the bone marrow [17]. As they mature, the multiple nuclei of the MK are produced by endomitosis [17]. After becoming multinucleate, MKs then begin cytoplasmic maturation [17] during which they produce a membrane demarcation system, which is an extension of their cell membrane that invaginates into the cell. This forms the production centre for platelets [18]. Proplatelet extensions or large fragments of MKs emanate from the membrane demarcation system into the bone marrow sinusoids, where the blood flow buds off nascent platelets from the tip of each or breaks up larger fragments of MKs [19,20] (Figure 2). Each MK is estimated to produce 100–1000 platelets and the collective production of platelets is estimated at 1–2 × 10^11^ each day [17,19]. Platelets survive for 7–10 days in the circulation, before being removed by the spleen [17].

## Producing megakaryocytes *in vitro*

MKs are found in very small numbers in the bone marrow, thus they cannot be harvested and transplanted from one person to another. They differentiate from HSCs, which are also present in small numbers the bone marrow. HSCs can be mobilised from peripheral blood or harvested directly from the bone marrow or from cord blood [21,22]. HSCs can be transplanted, but they generate the entire blood system and we have no way of directing their differentiation solely towards MKs *in vivo*. Therefore, to generate universal platelets, rather than transplanting MKs or their progenitors, we need to produce MKs *in vitro* and use these for the production of platelet units which we can then transfuse.

HSCs can be differentiated with a cytokine cocktail to form mature MKs which are capable of platelet production [23]. However, HSCs cannot be expanded *in vitro* and their MK production efficiency falls well below that required for the generation of clinically relevant platelet numbers. In addition, HSC isolation requires either invasive bone marrow sampling or the treatment of donors with granulocyte colony-stimulating factor for several days before donation. It would be beneficial to turn towards a cell type which could be easily generated and expanded *in vitro*.

Induced pluripotent stem cell (iPSC) lines represent a possible alternative source of seed cells. While overcoming the potential ethical issues associated with human embryonic stem cells (hESCs), iPSCs can produce all differentiated cell types and are infinitely expandable *in vitro*, overcoming the need to repeatedly harvest primary cells. They can also be gene-edited and quality-controlled, generating HLA Class I knockout (KO) lines for the generation of universal platelets.

**Figure 3.**
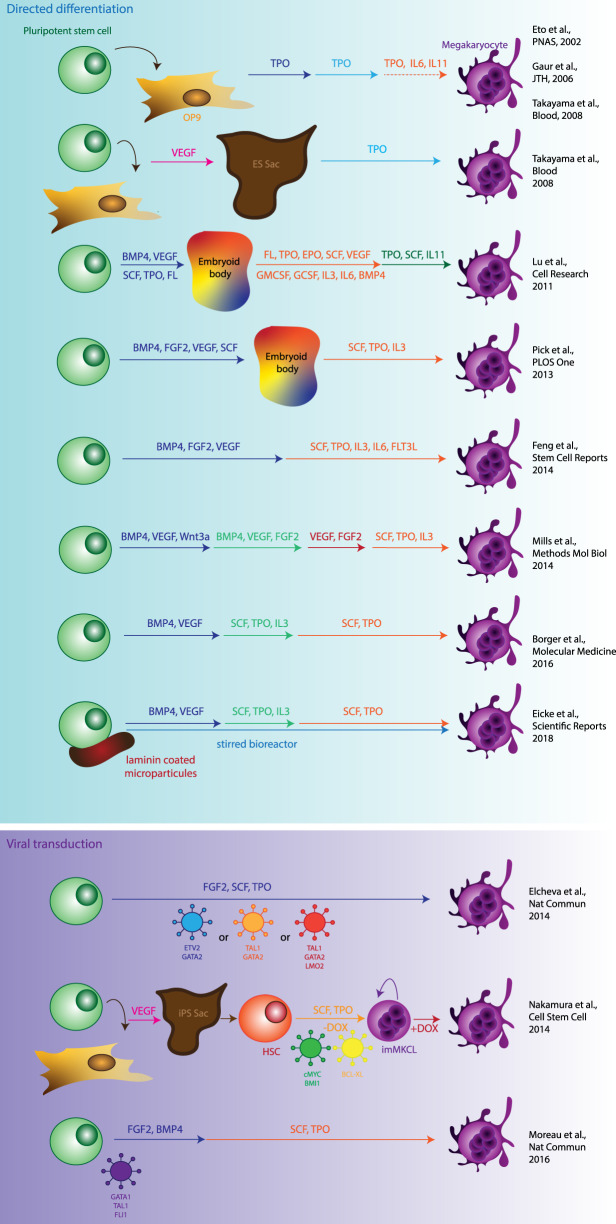
Summary of *in vitro* MK differentiation protocols. Schematic of optimised protocols for the differentiation of MKs from pluripotent stem cells, either by directed differentiation using cytokines alone or with the aid of transgenes in addition to cytokines.

There are several systems which have been developed for the production of MKs from pluripotent stem cells (Figure 3). First of all, cytokine cocktails [invariably containing thrombopoietin (TPO)] can be applied to pluripotent cells to mirror MKs’ *in vivo* developmental trajectory and shepherd the cells towards MK differentiation [24–26]. This is known as ‘directed differentiation’. Directed differentiation can be aided by feeder layers such as OP9 stromal cells [27,28] upon which embryonic stem cells (ESCs) produce sac-like structures [29]. These sac-like structures contain a large amount of progenitors which can differentiate into mature MKs. Output can be increased by the treatment of differentiating sacs with VEGF (vascular endothelial growth factor) [29].

Progressing away from feeders and undefined conditions, MKs were then differentiated from hESCs using TPO, SCF and IL11 in defined media conditions [30]. These MKs were produced in large numbers at around 118 per starting iPSC and could also produce platelets that contributed to clot formation in a mouse model. A second group also generated MKs from hESCs without feeders in defined conditions using an embryoid body-based protocol [31]. Interestingly, these could also fragment to generate platelet-like particles.

Directed differentiated methods alleviate the risk to patients from transduced or transformed cell products, as the cells are unmodified [24]. Directed differentiation approaches can also be combined with the use of bioreactors, as the human pluripotent stem cells (hPSCs) can be loaded onto laminin-coated microcarrier beads and the cell suspension stirred as they differentiate into MKs. This can increase output per starting hPSC [4]. However, the cytokine dosage required for these protocols remains high, increasing the production costs of any potential cellular product. In addition, directed differentiation protocols are still often hampered by low production efficiency.

Because of this low efficiency, groups aimed to increase the efficiency of MK differentiation with viral transgenes. Platelet release from *in vitro*-derived MKs was increased after transduction with CMYC [32]. Thus, the idea of directing cell fate with viral transgenes began to gain a foothold. Another group generated cells with MK erythroid progenitor-associated surface marker expression after transducing hESCs which GATA2, TAL1 and LMO2 [33]. Transducing ESCs with ETV2 and GATA2 generated cells which could form MK-like colonies in colony forming assays [33].

The Eto group then extended their ES-Sac protocol to include viral transgenes. After the generation of the sac-like structures, haematopoietic progenitor cells were isolated and immortalised by viral transduction with CMYC, BMI1 and BCLXL, enabling expansion for up to 5 months after transformation [34]. Upon removal of the viral transgenes, the cells matured and produced platelets, demonstrating that despite the immortalisation, the MKs produced remain functional. However, an important caveat of this approach is the potential for tumorigenesis. The authors observe that after prolonged culture, some clones acquired karyotypic anomalies which could generate leukaemia in recipient mice. Because the safety controls in place for the production of platelets *in vitro* would probably involve irradiation of the cells before transfusion and BMI1 overexpression could rescue cells from apoptosis [35], this presents a possible safety concern.

Work in our laboratory has generated a protocol for the generation of expandable MKs cultures without the need for transformation [36]. This is achieved by the transduction of hiPSCs (human-induced pluripotent stem cells) with GATA1, TAL1 and FLI1. Within 20 days in only two cytokine combinations, these cells generate a pure population of mature MKs which can produce platelets *in vitro* [5]. These cultures can be maintained for up to 120 days in culture without losing their platelet production abilities and can be frozen and rethawed.

## Bioreactors for the generation of platelets from *in vitro*-produced megakaryocytes

After the generation of mature MKs from hPSCs, the MKs need to be fragmented to form platelets. *In vivo*, this is done as or after the MK approaches the lumen of the bone marrow sinusoids and buds through into the circulation. Various models have been proposed through which MKs form platelets [17,19,20], however, the consensus in the field would suggest that blood flow through the bone marrow drives the disruption of the MK and the formation of platelets [17,20,37]. To achieve this *in vitro*, bioreactors have been designed to mimic the shear the blood flow would exert on MKs *in vivo*. These vary significantly in design. A recent publication by the Eto group describes a large stirred bioreactor which uses the vertical movements of large paddles to fragment mature MKs [38]. Microfluidic devices have also been designed which adhere MKs to a fenestrated membrane using fluid flow [39]. Their membranes are then forced through the fenestrated membrane and budded off, forming platelets. A third method of deriving platelets from their parent MK involves loading a sponge of defined pore sizes with the mature MKs [5,40]. Fluid can then be pumped through the sponge to encourage the MKs to release platelets, which due to their smaller size can flow freely out of the sponge. Finally, a fourth model has been designed which loads the MKs onto a silk sponge fitted with multiple narrow channels, mimicking intramarrow blood vessels. MKs adhere to the multiple channels and bud off their platelets in response to media flow [41]. Almost universally, thus, these bioreactors utilise fluid flow to produce platelets. Additionally, the sponge systems are amenable to functionalisation in order to mimic the chemical environment of the bone marrow. Most of the systems, apart from the first, also separate the parent MKs from their daughter platelets, ensuring the lack of nucleated cells in the final product. In the first system, the separation between platelets and MKs is done by centrifugation which may adversely affect the platelets’ quality as a transfusion product.

## HLA Class I deletion

The HLA system is a series of closely linked genes which together are responsible for the presentation of antigens to the immune system and immune surveillance of the body [42]. HLA Class I genes include the classical HLA-A, -B and -C, the non-classical HLA-E, -F and -G and MHC Class I chain-related MICA and MICB. Classical HLA Class I proteins are expressed on most nucleated cells whereas the non-classical and associated proteins have more restricted expression ranges [42]. HLA Class I presents antigens to CD8^+^ cytotoxic T lymphocytes. HLA-A, -C, -E and -G can also present to natural killer (NK) cells, γδ T cells and CD8^+^ T cells. Interestingly, most anti-platelet antibodies generated by patients who become refractory to transfusion are against HLA-A and -B [9]; thus, these represent a possible target for deletion with an aim to the transfusion of at-risk patients.

For correct assembly on the cell surface, HLA Class I requires β2-microglobulin. To delete all HLA Class I without targeting all the genes separately, β2-microglobulin can be disrupted. This was first carried out by the lentiviral transduction of iPSCs with shRNA [26]. This resulted in an 87% reduction in the transcript levels of β2-microglobulin without compromising the cells’ expression of pluripotency-associated markers. These cells were then differentiated into MKs by three sequential cytokine cocktails. Differentiation efficiency was not adversely affected by the reduction in HLA Class I. Furthermore, the platelets produced from these HLA Class I-deficient MKs were functional and survived longer in a mouse model for platelet refractoriness than control platelets. This was the first indication that HLA Class I deletion is both possible and beneficial for human *in vitro*-produced platelets. However, with this system, HLA Class I levels were reduced but the protein was not completely absent. The absence of HLA Class I is reported to cause cytotoxicity by the activation of NK cells [43]. Therefore, it was not clear whether the minimal residual levels of HLA Class I were sufficient to block this destruction.

HLA Class I knockdown (KD) has also been carried out in CD34^+^ stem cells in order to generate HLA Class I KD MKs [44,45]. Again, this was carried out by transduction of the cells with a lentivirus encoding anti-β2-microglobulin shRNA. These CD34^+^ cells could then be differentiated into MKs, interestingly at higher efficiency than controls [45]. They also generated higher numbers of platelets. Crucially, HLA Class I KD cells could evade antibody-dependent cellular cytotoxicity *in vitro*, indicating that they might escape immune surveillance better than control cells [44]. To investigate the immune response to these cells *in vivo*, the group then generated a mouse model for platelet survival. MKs from KD and control CD34^+^ cells were then injected into NOD/SCID/IL2RγC^−/−^ (NSG) mice. Both KD and control MKs could produce platelets *in vivo*, however, upon the addition of human anti-HLA Class I antibodies, control platelets were destroyed, whereas HLA Class I KD platelets survived at higher levels for up to 11 days [44]. This demonstrates the utility of HLA Class I deletion or reduction for transfusion, as it could enable the survival of transfused platelets in the circulation, despite circulating antibodies against HLA Class I. However, NSG mice will not have human NK cells, and this poses a problem for the proper assessment of NK-mediated destruction of HLA Class I deleted cells. HLA Class I expression in the CD34^+^ cells used in these studies was 85% of control levels, thus residual protein levels could inhibit the activation of NK cells if the cells were exposed to a full human immune system [45]. The levels of HLA Class I which are required to appease NK cells remained to be tested.

To address this, the authors then turned to a model rat system. They silenced rat HLA Class I in fibrosarcoma cells by transducing them with lentivirus encoding shRNA against β2-microglobulin [46]. These were then transplanted into rats. Cells which had silenced HLA Class I formed larger tumours than control cells, illustrating the idea that HLA Class I removal enables cells to evade immune recognition. HLA Class I KD tumours were infiltrated with less CD4^+^ and CD8^+^ T cells than controls, indicating that the cells elicited poorer lymphocyte activation, which they also demonstrated using a T-cell proliferation assay. Interestingly, HLA Class I silenced tumours did not have increased NK cell infiltration, indicating that the reduction in HLA Class I does not activate the ‘missing self’ immune response [46,47]. *In vitro* assays also suggested that NKs do not recognise fibroblasts expressing more than 10% of the normal levels of HLA Class I and as these cells had 15% HLA Class I expression remaining, they could escape NK cell recognition.

To mitigate the risk that HLA Class I null cells could be destroyed by NK cells, it could be useful to express cell surface receptors which activate inhibitory NK receptors. For example, HLA-E binds to NKG2A/CD94 and protects HLA-E-expressing cells from NK-mediated lysis [48–50]. Human HLA-E was expressed in pig cells, which then protected them from lysis *in vitro* by human NK cells, indicating that HLA-E overexpression could be a complementary strategy to protect HLA-A, -B and -C null cells from NK-mediated lysis [51]. HLA-G overexpression has also been carried out in hESCs and this also protected ESC-derived epithelial progenitors from NK-mediated lysis *in vitro* [52]. However, HLA-E and -G require β2-microglobulin for assembly of their membrane-bound forms, therefore, using these immune modulators in HLA Class I KO cells would require β2-microglobulin re-expression, fused to HLA-E or -G [48,53,54].

As gene editing techniques advanced, it became more straightforward to produce β2-microglobulin KO cells, which could then be used for the differentiation of MKs. This was first done in 2014, where the authors used TALENs to KO β2-microglobulin by the disruption of exon 2 [24]. A cytokine-mediated directed differentiation approach was then used to differentiate β2-microglobulin^−/−^ iPSCs into MKs. The platelets remained HLA Class I null and were activated in response to thrombin to a similar extent to controls [24]. This indicates that HLA Class I null platelets can be produced and remain functional. However, it remains to be demonstrated whether these HLA Class I null cells are recognised by the human immune system and specifically whether they might be targeted for destruction by NK cells. To address this, human HLA Class I KO platelets should be assessed in a humanised mouse model which would enable the interaction of *in vitro*-derived platelets with human NK cells *in vivo* [55]. This would be the first step towards the generation and transfusion of universal platelets.

## Quality control of edited cellular products

**Figure 4.**
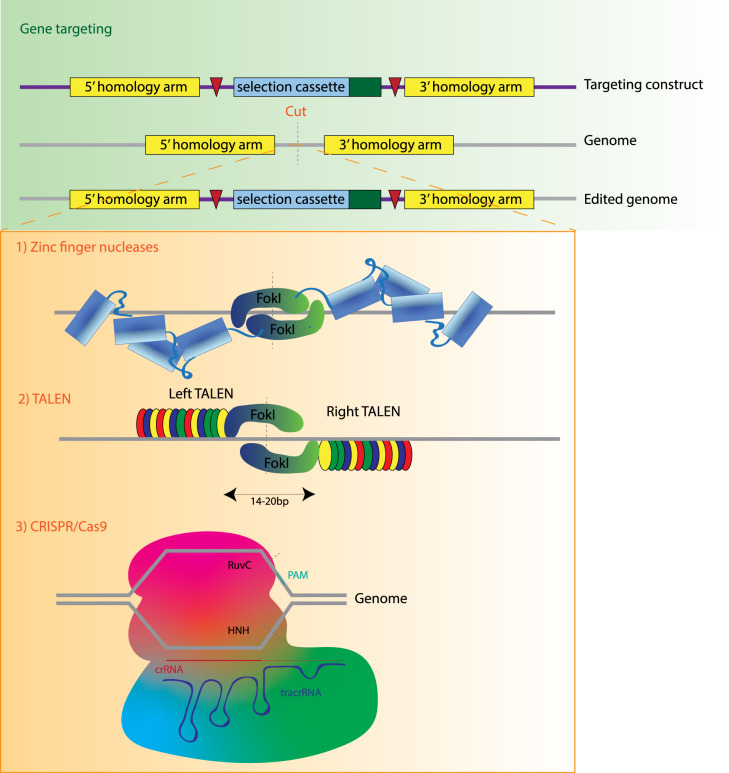
Methods of gene editing. Gene editing methodologies with possible uses to generate universal platelets. Gene targeting is shown in the green panel, which is inefficient in most cell types and requires the generation of DNA breaks. Nucleases which can generate these targeted breaks are shown in the orange panel.

The advent of genome engineering also creates a new landscape for the regulation and quality control of cellular products. Cells edited with CRISPR/Cas9 need to be assessed for safety before approaching clinical trials. Gene editing systems (Summary in Figure 4), as well as the clonal selection necessary to start master cell banks of edited iPSCs can introduce potentially deleterious mutations into pluripotent stem cells. For example, it has recently been demonstrated that hPSC cultures can acquire p53 mutations during extended culture [56]. These mutations may allow cells to outcompete their neighbours, eventually taking over the culture [57].

To address these concerns, strict quality control must be carried out on the starting cell population and repeated at regular intervals during differentiation. Current work in the field is addressing which techniques may be best for efficient and sensitive quality control. Karyotyping may be useful to identify large abnormalities, whereas qPCR of key loci, fluorescence *in situ* hybridisation and droplet PCR could be useful to identify the emergence of smaller mutations [58]. Combining this with regular analysis of cancer-associated single nucleotide polymorphisms by SNP array could enable the early detection of cancer- and proliferation-associated mutations [59]. Pluripotent starting cells should be subjected to a highly stringent quality control step, including not only karyotyping but more detailed analyses analysing genome integrity such as SNP arrays, FISH or whole-genome sequencing analysis. The final MK product should also be strictly controlled, possibly including SNP arrays, karyotype, clonogenicity assays and the analysis of a panel of markers by flow cytometry.

In addition, there is much concern about potential off-target effects of CRISPR/Cas9, many of which can be mitigated by the use of paired Cas9 nickases [60] and the use of proteins instead of nucleic acids [61] but some of which may still be of concern. The starting pluripotent stem cell also needs to be available at clinical grade, posing an additional hurdle to line selection for differentiation [62]. Finally, the transplant or transfusion of a human recipient with an edited cellular product represents an enormous safety concern for regulators, particularly where the cell type or its progenitor or stem cell is long-lived and has the potential to colonise the body or generate tumours. Cells should be terminally differentiated and efficiency should be regularly analysed, to exclude proliferating progenitors from any potential cellular product. Additional layers of security can also be built into the system by the use of suicide genes. For example, iPSCs can be transduced with a caspase-9 which is expressed by treating cells with a chemical inducer [63]. Tumours from the cells can then be eradicated by treating cells with the chemical inducer. Crucially, this is effective *in vitro* as well as *in vivo*, providing a rapid method to remove any escaping cells, if necessary.

In conclusion, quality control of both the starting pluripotent cell type and the final differentiated cell product are critical steps in providing a safe and consistent product. Much work remains to be done on determining which quality controls need to be put in place and the sooner industry standards are set, the sooner *in vitro* platelets can progress towards the clinic. Many cellular products are already well advanced towards clinical trial [64] and defining quality control standards remains a critical step in this process.

## Perspectives

Work by multiple groups has demonstrated the robust and reproducible differentiation of mature MKs from many cell sources including CD34^+^ stem cells from peripheral blood, hESCs and hiPSCs. MKs have also been transdifferentiated from fibroblasts [65]. Progress has also been made towards the reduction or deletion of β2-microglobulin with a view to generating platelets with reduced or absent HLA Class I. However, it still remains to be addressed whether *in vitro*-derived HLA Class I KO platelets can survive the human immune system, and furthermore whether HLA Class I deletion is the best path towards a universal transfusion product [66]. Because they degranulate at the site of injury, platelets represent an unrivalled method of targeting drugs after haemorrhage or infarction. The systems we use to generate them also provide us with a key insight into processes which occur deep in the bone marrow during MK maturation. Analysis of differentiating MK cultures can enable us to understand megakaryopoiesis *in vivo* and take steps towards ameliorating bleeding disorders or deficiencies in MK maturation in patients. The path towards the clinic is a shorter one for platelets than for many other cellular products; however, much work still needs to be done before we can generate universally transfusable platelets.

## Summary

- Megakaryocytes can be reproducibly and efficiently differentiated from human pluripotent stem cells, enabling us to produce platelets *in vitro*.
- The knockdown or deletion of β2-microglobulin can generate HLA Class I knockdown or knockout MKs, which can produce platelets for universal transfusion.
- Much optimisation is taking place on methods of differentiation, with a particular aim to decrease cytokine requirements, increase the generation of mature MKs and reduce culture volume for the production of platelet units.
- Standard quality control procedures are needed for the production of cellular products and the control of gene editing effects need to be factored into all cellular therapy production protocols.

## Abbreviations

ESCs: embryonic stem cells
hESCs: human embryonic stem cells
HLA: histocompatibility leukocyte antigen
hPSCs: human pluripotent stem cells
hiPSCs: human-induced pluripotent stem cells
HSCs: haematopoietic stem cells
iPSC: induced pluripotent stem cell
KD: knockdown
KO: knockout
MK: megakaryocytes
NK: natural killer
TALENs: transcription activator-like effector nucleases
TPO: thrombopoietin
